# Influence of File Motion on Shaping, Apical Debris Extrusion and Dentinal Defects: A Critical Review

**DOI:** 10.2174/1874210601812010189

**Published:** 2018-02-28

**Authors:** Victor Feliz Pedrinha, Juliana Melo da Silva Brandão, Oscar Faciola Pessoa, Patrícia de Almeida Rodrigues

**Affiliations:** School of Dentistry, Federal University of Pará, Pará, Brazil

**Keywords:** Endodontic files, Endodontically treated teeth, Kinematics, Nickel-titanium, Root canal preparation, Root canal therapy

## Abstract

Advances in endodontics have enabled the evolution of file manufacturing processes, improving performance beyond that of conventional files. In the present study, systems manufactured using state of the art methods and possessing special properties related to NiTi alloys (*i.e*., CM-Wire, M-Wire and R-Phase) were selected. The aim of this review was to provide a detailed analysis of the literature about the relationship between recently introduced NiTi files with different movement kinematics and shaping ability, apical extrusion of debris and dentin defects in root canal preparations. From March 2016 to January 2017, electronic searches were conducted in the PubMed and SCOPUS databases for articles published since January 2010. *In vitro* studies performed on extracted human teeth and published in English were considered for this review. Based on the inclusion criteria, 71 papers were selected for the analysis of full-text copies. Specific analysis was performed on 45 articles describing the effects of reciprocating, continuous and adaptive movements on the WaveOne Gold, Reciproc, HyFlex CM and Twisted File Adaptive systems. A wide range of testing conditions and methodologies have been used to compare the systems. Due the controversies among the results, the characteristics of the files used, such as their design and alloys, appear to be inconsistent to determine the best approach.

## 1 INTRODUCTION

Nickel-titanium (NiTi) files were introduced in the manufacturing process in 1988 [1] to avoid unwanted changes in the morphology of root canals caused by stainless steel files. These files have shown a significant increase in flexibility, enabling the development of rotational automated systems and improving efficiency in root canal shaping [2].

Recently, a thermomechanical procedure appropriate for nitinol, an alloy component, aiming to produce super-elastic NiTi wires that can be used to manufacture endodontic files with improved fatigue resistance [3] was developed; the wires are collectively called M-Wire NiTi (Dentsply Tulsa, OK, USA). VDW (Munich, Germany), the company that produces the Reciproc system, uses M-Wire technology to manufacture NiTi files that use reciprocating movements [4]. The WaveOne Gold system (Dentsply Maillefer, Ballaigues, Switzerland) maintains the same file as the WaveOne reciprocating system but has modified dimensions and geometry. The file has a parallelogram-shaped cross-section with 2 cutting edges. The manufacturing process consists of a thermal treatment performed repeatedly by heating and slow cooling, resulting in a golden file [5].

Another change in NiTi alloys has been developed to increase the efficiency and flexibility of rotating files, as occurs in the files of the HyFlex Controlled Memory (CM) system (Coltene Whaledent, Cuyahoga Falls, OH, USA), containing a smaller percentage of nickel than other systems [6]. The reduction of nickel content generates a metal that is softer, *i.e*., exhibits lower hardness [7]. The processing of these files also affects the metal properties, such as the thermal changes that occur during the manufacturing of the HyFlex CM file, which results in a martensitic metal phase. The martensitic phase is a more flexible form of yarn that results in greater elasticity and resistance to cyclic fatigue [8].

In the adaptive motion Twisted File Adaptive (TFA) system (Sybron, Orange, CA, USA), the NiTi alloy is manufactured by R-Phase technology. This system presents a lower degree of apical transport and greater centralization of the canal; in addition, the system maintains the path in the apical third of severely curved proximal surface canals [9].

Because the kinematic effects of preparation systems affect the quality of endodontic treatment, a system in which better clinical results are obtained through biomechanical preparation techniques is expected. Advances in endodontics have enabled the evolution of file manufacturing processes, improving performance beyond that of conventional files. In the present study, systems manufactured using state of the art methods and possessing special properties related to NiTi alloys (*i.e*., CM-Wire, M-Wire and R-Phase) were selected. The aim of this study was to review the articles including *in vitro* studies performed only in extracted human teeth and involving automated systems with files produced by NiTi alloys found in the Reciproc, WaveOne Gold, HyFlex CM and Twisted File Adaptive systems and their possible differences with respect to shaping ability and formation of apical debris and dentinal defects.

## 2 SEARCH METHODOLOGY

Between March 2016 and January 2017, 2 independent researchers searched the electronic bibliographic databases MEDLINE/PubMed and SCOPUS (ELSEVIER) for *in vitro* studies performed on extracted human teeth (in English language). The study was complemented with a search of citations from relevant articles on the subject. Searches in the MEDLINE/PubMed database did not have filters or limits applied; filters were used to restrict the searches in SCOPUS (ELSEVIER) to articles concerning dentistry.

The electronic searches resulted in the identification of 736 articles (396 from PubMed and 340 from SCOPUS), which were exported to the Mendeley Desktop software program in database groups. The abstracts were first screened to eliminate articles that clearly failed to meet the search criteria. Based on the inclusion criteria, 71 papers were selected for the analysis of full text copies. Specific analysis was performed on 45 articles, describing the effects of reciprocating, continuous and adaptive movements on the WaveOne Gold, Reciproc, HyFlex CM and Twisted File Adaptive systems. The articles were required to describe *in vitro* studies on automated systems with respect to one of the following subjects: shaping, the apical extrusion of debris or the formation of dentinal cracks.

The exclusion criteria pertained to publication type and excluded reviews, editorials, opinions, technical articles, guidelines, comment articles, animal studies, artificial or simulated canals, studies on deciduous dentition, and studies evaluating systems through endodontic retreatment. Combinations of the terms used in the search are described in Figs. (**1**-**3**).

From selected studies, the following data were extracted and included in the review: sample size or type, type of files used, kinematics, and methods used for obtaining and evaluating results (shaping ability, amount of apical debris and dentinal cracks).

## 3 LITERATURE REVIEW

The literature review was organized into three sections: (I) comparative shaping ability for canal transport; (II) comparative studies of apical extrusion of debris; and (III) comparative studies of the formation of dentinal defects, featuring biomechanical preparations carried out by automated systems. The types of movement were investigated in these studies, and their characteristics are described in Tables **1**, **2** and **3**.

## 4 SHAPING ABILITY

One of the main objectives of biomechanical preparation is to promote the modeling of root canals, ensuring conicity with crown-apex tapering, such that the original shape of the canal is maintained. However, in curved canals, procedural errors such as apical transport and zips are common during this stage [10]. The elasticity of NiTi rotary files allows clinicians to conduct the desired form of taper root canal therapy with a reduced tendency for transport [11].

Based on peer-reviewed articles, it can be affirmed that all publications used curved canals in laboratory experiments; the angle of curvature of the roots varied between 10 and 45 degrees in most proximal surfaces of the molars. With respect to the efficiency of systems and the techniques developed for preparing canals and determining apical transports, several methods were used to compare the shape of the canals before and after preparation, such as the radiographic overlay method, cone beam computed tomography (CBCT) and micro-computed tomography (µCT).

Among the studies that showed significant differences in transport values, comparative analyses before and after preparation were mostly performed by the µCT evaluation method [9, 12-15], followed by studies that used CBCT [16-19] and finally, by studies that used radiographic evaluation methods pre- and post-instrumentation through computer image analysis programs [20, 21]. The high precision of three-dimensional (3D) analysis of root canal systems in experimental procedures is significant in justifying image comparison by µCT as the more sensitive methodology for measuring transport values. In endodontic studies, µCT analysis is performed by direct comparison of canal system images before and after biomechanical preparation through computer programs that are capable of presenting 2D and 3D morphological data of the root canals [22].

In the 5 articles [23-25] that were reviewed, kinematics showed no statistically significant differences in relation to transport values in the apical third; in 3 of the articles, no statistically significant differences were observed between continuous and alternate rotation kinematics. One article showed no significant difference about transport in the apical third between systems of continuous rotation and adaptive motion [26]; furthermore, no significant differences between the 3 types of kinematics were observed [27].

Four articles involving systems with alternate motion presented results indicating greater transport values than those of continuous rotation systems [12, 14, 17, 21]; in contrast, 5 articles [15, 16, 18-20] indicated greater transport values for continuous systems than for alternate motion systems. Regarding the articles that demonstrated higher values of apical transport for systems with alternating rotation, teeth treated with the Reciproc system were associated with significant increases in the area, perimeter and diameter of the main canal relative to systems of continuous rotation and adaptive movement. Some authors related the design of the file to the cutting ability of dentinal walls, particularly for the Reciproc system, which presents an S-shaped cross-section and sharp cutting edges. The design of the file together with the reciprocating movement increases the cutting efficiency of Reciproc files. In these articles [12, 14, 17, 21], root canals were modeled by selected files with similar sizes and tapers in the different comparative groups (file size 25 and 0.08 taper).

On the other hand, based on the results obtained in articles concerning apical transport, the smaller transport values obtained for alternate movement systems compared with continuous motion systems (*P* < 0.05), particularly for the Reciproc system, can be justified by the movement performed at rotation angles of 150° counterclockwise and 30° clockwise; because the rotation in the direction of the cut is greater than that in reverse, the file moves toward the apex. This reciprocal motion decreases the pressure on the tool and reduces the risk of cyclic fatigue caused by tension and compression, in addition to maintaining the centralization of the canal [15, 16, 18-20]. Moreover, M-Wire technology confers greater flexibility to these files because of the low modulus of elasticity [28].

Two articles [9, 13] compared alternate and adaptive systems; lower values were obtained for the TFA system. According to Gergi *et al.* [9, 13], the TFA system presented the lowest transport values in 2 articles that comparatively evaluated the Reciproc and WaveOne reciprocating systems in addition to the TFA adaptive system by µCT. The TFA system uses a combination of continuous rotation and alternate movements, in which continuous rotation is used when the amount of pressure exerted on the file is minimal and reciprocal movement is used when the dentin has an applied load. The manufacturers claim that this adaptive technology and twisted file design increases flexibility and allows the file to adjust torsion forces inside canals, depending on the amount of pressure that is exerted. The adaptive movement is based on a patented algorithm that produces changes in motion in accordance with the loads that are applied to the file through the walls of the canal. This patented algorithm adapts to different conditions based on the amount of pressure on the file when it is not under pressure and will rotate continuously clockwise without anticlockwise motion, but when the file is subjected to pressure loads, the alternating angles vary, 370° clockwise and 20° to 50° counterclockwise, based on the load applied to the file [9, 13, 29].

With an apical preparation diameter similar to the values reported in other studies, the TFA system demonstrated that it could produce significantly less transport, followed by the WaveOne and Reciproc systems. The combination of adaptive movement and the flexibility of the files is proposed as the reason for the modeling results obtained for the TFA system [30].

## 5 APICAL EXTRUSION OF DEBRIS

During root canal shaping, dentin scrapings, pulp tissue fragments, necrotic tissue, and microorganisms or irrigating agents can be eliminated from periodontal tissues [31]. Apical extrusion of infected debris may have the potential to disrupt the balance between microbial aggression and protection of the host, resulting in episodes of periapical inflammation and flare-ups after surgery [32]. While all staging techniques cause apical extrusion of debris, the amount of debris extrusion in the periapical region may vary depending on the method used [33].

All articles examined in this review presented experiments involving the apical extrusion of debris. Two articles did not show statistically significant differences regarding the comparative amounts of debris in reciprocating systems in evaluating continuous rotation in lower premolars [34] and adaptive movement in mandibular incisors [35].

Five articles obtained significant differences using anterior teeth; in 2 of these articles, the Reciproc system resulted in a greater amount of debris than that generated by continuous rotational systems [36, 37]. Another article he first 3 mm of the workpiece, which decreasshowed that continuous systems produced a greater amount of debris than reciprocating systems such as Reciproc and WaveOne [38]. Two articles used only one type of file in 2 alternate and continuous rotational kinematic mechanisms with the Reciproc and TFA systems [39, 40]. According to Ahn *et al.*, these two studies excluded the effect of file design, indicating controversial results [41].

With respect to premolars, 8 articles showed significant differences in their results. In only 1 article [40] addressing apical debris, the WaveOne Gold (WOG) system presented better results than the conventional WaveOne (WO) system and continuous rotation systems such as the ProTaper and ProTaper Universal Gold systems. Because of the absence of further studies on WOG, performing a comparative analysis is difficult; however, some differences between WOG and WO files should be highlighted.

WOG “primary” files have a taper of 0.07, while primary files in a conventional WO system have a taper of 0.08. This discrepancy may be responsible for the greater flexibility of WOG files presenting a parallelogram design with one or two cutting edges depending on the location along the file. The WO system presents a cross-sectional triangular design that can result in a smaller screwing effect and higher cutting efficiency. These differences in cross-section, *i.e*., the taper and the flexibility of the files, may be responsible for the low amount of apical debris generated by WOG files [40].

The HyFlex CM system showed low amounts of extrusion of debris in reciprocating systems in 2 articles [42, 43]; this finding can be explained by the HyFlex CM system’s slightly convex triangular cross-section, while the Reciproc system exhibits higher cutting efficiency [42]. Articles indicate that differences in conicity between files may influence debris extrusion [42, 43]. Reciproc R40 (0.06 taper) and WaveOne Large (0.08 taper) files have a constant taper within the first 3 mm of the workpiece, which decreases to *D*16, while the HyFlex CM system has a continuous taper of 0.04. The greater contact at the tip of the WaveOne and Reciproc files may promote greater extrusion of debris compared with that of the HyFlex CM system due to the greater wear of the dentin walls, which was not observed in the abovementioned articles [42, 43]. However, compared with the adaptive motion TFA system, the HyFlex CM files showed more debris extrusion in which deformities were observed on all files used for up to 3 single canals, which after the preparation, 80% of the HyFlex CM files were deformed. Another condition pointed as the cause of debris extrusion, was the files deformation, leading to the unwinding of their spirals, as observed in the HyFlex CM system [44].

Two articles compared continuous rotation systems with the WaveOne and Reciproc alternating systems and demonstrated lower extrusion about reciprocating or alternating files [45, 46]. Topçuoglu *et al.* [47] and Nayak *et al.* [48] indicated that the Reciproc system results in more extrusion of debris than some continuous rotation systems.

Three articles on molars were selected for this review. In these articles, the Reciproc system showed lower apical extrusion of debris [49, 50]; however, in the study by Kuştarcı *et al.*, there was no statistically significant difference between the Reciproc and TFA systems evaluated in premolars [51].

Because the number of *in vitro* studies is limited and many have submitted conflicting results, more studies are required. The WOG system suggests efficiency, but only one manuscript has addressed the subject to date. HyFlex CM files have demonstrated less extrusion than Reciproc files; however, the TFA system suggests better performance than HyFlex CM files. Reciproc files showed lower extrusion in six studies, while in four others, these results were contradicted.

## 6 FORMATION OF DENTINAL DEFECTS

Dentinal defects can propagate to a vertical root fracture and, in most cases, lead to tooth extraction. These fractures have a multifactorial etiology, and some authors attribute this condition to excessive biomechanical preparation, excessive removal of dentin during the widening of the canal and after preparation, the amount of remaining coronal structure, types of parafunction and excessive force during the filling of the root canal [52].

One can speculate that when using only a file for preparation, more stress will be generated during the instrumentation mechanics than when using canal preparation systems with string files. Thus, it is inferred that the incidence of defects can increase under these conditions compared with that observed in preparations using the complete sequence of rotational systems [53].

According to this review, 11 articles on dentinal defects were eligible; among these, 4 showed no statistically significant differences between continuous rotational and reciprocating systems [54-56]. One article showed no significant difference between a reciprocating system and the TFA system [57]. Only one article showed the absence of defects through image analysis by µCT [58].

The TFA system showed the best results in 3 articles when compared with only reciprocating systems [59] or with continuous systems and reciprocating systems [60, 61]. In 1 article [62], this system was evaluated by performing three types of kinematic assessments, in which alternating movement yielded better results with respect to the formation of defects in dentine. In these articles, adaptive movement is indicated as a cause of the decrease in the tension caused by the walls of the root canals, particularly in the apical third, resulting in less crack formation [59-62]. The variation in the taper of the files is also considered as a contributing factor in the formation of dentin defects. Karatas *et al*. [61], WaveOne Primary and ProTaper F2 files caused greater crack formation at the 3 mm level of the apical third. The WaveOne Primary file has a taper of 0.08 in its apical portion which decreases to 0.055 for the remainder of the working length. Likewise, ProTaper F2 has a taper of 0.08 in the apical portion. However, the ProTaper Next X2 and TF Adaptive SM2 files feature a taper of 0.06. The greater conicity of the F2 and WaveOne Primary files in the apical portion may have led to greater crack formation [61].

Two articles are controversial. Jalali *et al.* [63] observed lower defect formation in a dental group instrumented with the Reciproc system, while for the same system, Bürklein *et al.* [54], obtained less satisfactory results relative to those obtained for the Mtwo (Sweden & Martina, Padova, Italy) and ProTaper (Dentsply Maillefer, Ballaigues, Switzerland) systems with continuous rotation. Thus, in these cases again, the file taper and the kinematics employed by each of them, interfere to obtain different results.

## CONCLUSION

Various NiTi files with different designs produced by different manufacturing methods have been introduced in dentistry. These files provide many advantages over conventional files, such as increased flexibility and improved efficiency in root canal shaping. Considering the three categories reviewed (shaping ability, extrusion of debris, and dentinal defects or cracks), in comparing shaping results based on the apical transport value, µCT was determined to be the methodology that yielded the most significantly different results between the kinematic methods. With respect to shaping ability, the TFA system yielded the best results in 2 studies compared with reciprocating systems developed by the same author. For apical extrusion of debris in premolars, the WOG system presented better performance than the WaveOne system, which performs reciprocating kinematics, and the ProTaper Universal and ProTaper Gold systems, which perform rotational kinematics. The TFA adaptive motion system yielded better results than HyFlex CM files with respect to debris extrusion. In molars, the Reciproc system presented better results than continuous rotation systems. Regarding the shortcomings of the various systems, the TFA system presented lower values with respect to the formation of dentinal defects in three studies, with comparisons made between reciprocating and continuous systems. Because of the variation in the results, the characteristics of the files, such as their design and the wires used, appear to be inconsistent to determine the best approach. In addition, laboratory studies are limited when comparing the kinematics of the systems.

## Figures and Tables

**Fig. (1):**
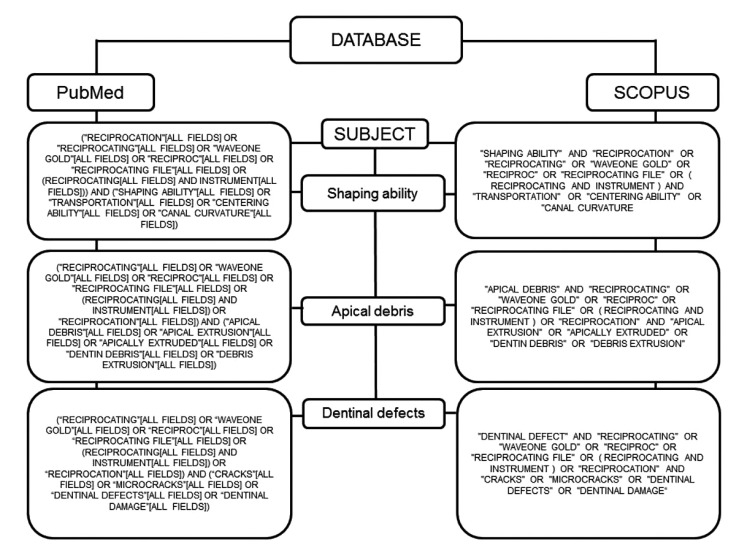
Combination of terms used for database search related to the WaveOne Gold and reciproc systems.

**Fig. (2):**
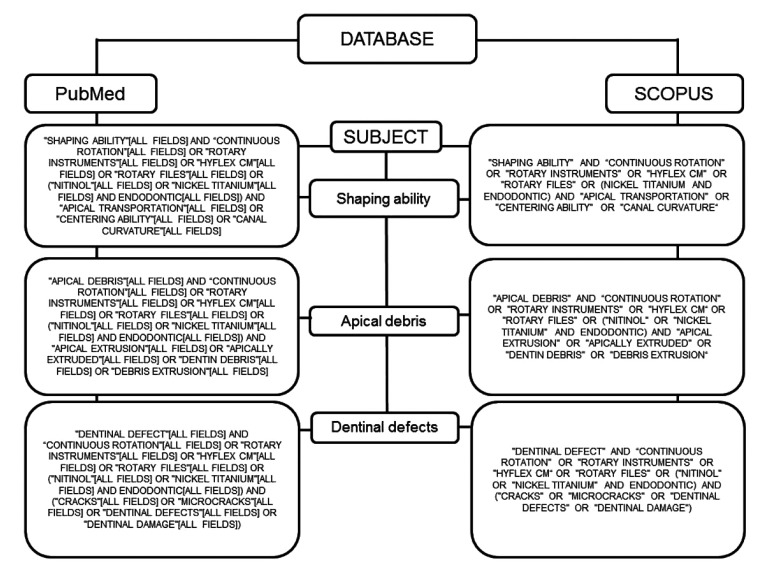
Combination of terms used for database search related to the HyFlex CM system.

**Fig. (3):**
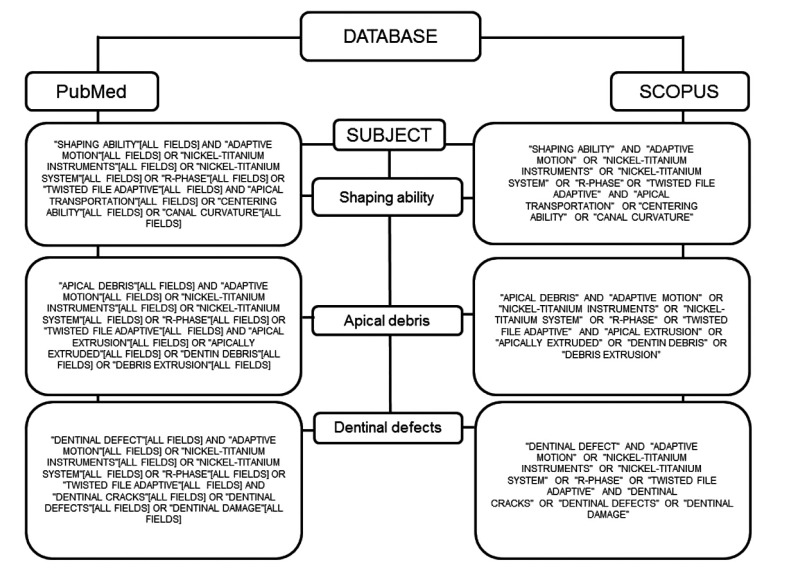
Combination of terms used for database search related to the Twisted File adaptive system.

**Table 1:** Summary of characteristics of the studies about shaping ability.

**Author**	**Type of Sample**	**Number of Canals**	**File Used**	**Kinematics**	**Methods**	**Results (Transportation)**
**Marceliano-Alves *et al.***	Mn molar 10º - 20º curvature	64	Reciproc WaveOne Twisted File HyFlex CM	R R CR CR	µCT	Reciproc and WaveOne > Twisted File and HyFlex CM
**Saber *et al.***	Mn molar 20º - 45º curvature	60	Oneshape Reciproc WaveOne	CR R R	Radiographic	Oneshape > Reciproc and WaveOne
**Simpsy *et al.***	Mn molar 25º - 35º curvature	45	HyFlex CM WaveOne ProTaper	CR R CR	CBCT	ProTaper > WaveOne > HyFlex
**Dhingra *et al.***	Mn molar (curvature not mentioned)	120	Oneshape Reciproc WaveOne	CR R R	CBCT	Oneshape > WaveOne and Reciproc
**Bürklein *et al.***	Human teeth (type not mentioned) 25º - 35º curvature	80	Mtwo Reciproc F360 OneShape	CR R CR CR	Radiographic	No significant difference
**Hwang *et al.***	Mx molar 20º - 45º curvature	45	Mtwo Reciproc	CR and R R	µCT	Mtwo RC > Mtwo R and WaveOne
**Bürklein *et al.***	Human teeth (type not mentioned) 25º - 39º curvature	80	Mtwo ProTaper Reciproc WaveOne	CR CR R R	Radiographic	No significant difference
**Ramazani *et al.***	Mn molar 20º - 40º curvature	64	K-File Mtwo Reciproc	MI CR R	CBCT	Mtwo > Reciproc
**Nabavizadeh *et al.***	Mx molar 25º - 35º curvature	60	Reciproc BioRace	R CR	Radiographic	Reciproc > BioRace
**Navós *et al.***	Mx molar 20º - 40º curvature	60	ProTaper MTwo Reciproc	CR CR R	CBCT	No significant difference
**Moazzami *et al.***	Mn molar 15º - 30º curvature	45	Neoniti Reciproc	CR R	CBCT	Reciproc > Neoniti
**Ahmetoglu *et al.***	Mx molar CV - 20º - 45º CP - 0º - 10º curvature	90	Self-Adjusting File Revo-S Reciproc	CR CR R	µCT	Reciproc > Self-Adjusting File and Revo-S
**Gergi *et al.***	Mn molar 25º - 35º curvature	48	Reciproc WaveOne Twisted File Adaptive	R R AM	µCT	Reciproc and WaveOne > Twisted File Adaptive
**Pedulla *et al*.**	Mn molar 25º - 35º curvature	64	Mtwo Twisted File Adaptive	CR and AM CR and AM	µCT	No significant difference
**Gergi *et al.***	Mn molar 25º - 35º curvature	48	Reciproc WaveOne Twisted File Adaptive	R R AM	µCT	Reciproc and WaveOne > Twisted File Adaptive
**Capar *et al.***	Mn molar 20º - 40º curvature	120	Oneshape ProTaper Universal ProTaper Next Reciproc WaveOne Twisted File	CR CR CR R R AM	CBCT	No significant difference

Mn, mandible; Mx, maxillar, VC, vestibular canal; PC, palatine canal; MI, manual instrumentation; AM, adaptive movement; CR, continuous rotation; R, reciprocation; CT, computed tomography; CBCT, cone beam computed tomography.

**Table 2:** Summary of characteristics of the studies about apical debris.

**Author**	**Type of Samples**	**Number of Canals**	**File Used**	**Kinematics**	**Irrigant**	**Results**
**Karatas *et al.***	Mn premolar	80	ProTaper Gold WaveOne Gold ProTaper Universal WaveOne	CR R CR R	Distilled water	ProTaper Universal > WaveOne, ProTaper Gold and WaveOne Gold
**Burklein *et al.***	Mn incisor	80	Reciproc F360 OneShape Mtwo	R CR CR CR	Distilled water	Reciproc > F360, OneShape and Mtwo
**Kucukyilmaz *et al.***	Mn premolar	45	Reciproc OneShape ProTaper ProTaperNext	R CR CR CR	Distilled water	No significant difference
**Nevares *et al.***	Mn premolar	60	Reciproc WaveOne HyFlex CM	R R CR	NaOCl	Reciproc > WaveOne > HyFlex CM
**Surakanti *et al.***	Mn premolar	60	ProTaper Hyflex WaveOne	CR CR R	Distilled water	WaveOne and ProTaper > HyFlex CM
**Capar *et al.***	Mn premolar	60	ProTaper Universal ProTaper Next Twisted File Adaptive HyFlex CM	CR CR AM CR	Distilled water	ProTaper Universal and HyFlex CM > ProTaper Next and Twisted File Adaptive
**Kirchhoff *et al.***	Mn incisor	48	ProTaper Next WaveOne Twisted File Adaptive	CR R AM	Distilled water	No significant difference
**Karatas *et al.***	Mn incisor	72	Twisted File Adaptive	R CR	Distilled water	R > CR
**Bürklein *et al.***	Mn incisor	80	Reciproc WaveOne Mtwo ProTaper	R R CR CR	Distilled water	WaveOne and Reciproc > Mtwo and ProTaper
**Arslan *et al.***	Mx incisor	45	Reciproc	R CR	Distilled water	CR > R
**De-Deus *et al.***	Mn molar	48	ProTaper WaveOne Reciproc	CR R R	Distilled water	ProTaper > WaveOne and Reciproc
**Cakici *et al.***	Mn molar	80	ProTaper Gold ProTaper Universal ProTaper Next Reciproc	CR CR CR R	Distilled water	ProTaper Universal and HyFlex CM > ProTaper Gold and Reciproc
**Uzun *et al.***	Mn premolar	60	WaveOne Reciproc SafeSider Typhoon ProTaper Universal Mtwo	R R R CR CR CR	Distilled water	Mtwo, ProTaper Universal, Typhoon, SafeSider and WaveOne > Reciproc
**Lu *et al.***	Anterior teeth (type not mentioned)	80	Reciproc WaveOne BLX ProTaper	R R CR CR	Saline solution	BLX and ProTaper > Reciproc and WaveOne
**Nayak *et al.***	Mn premolar	60	Reciproc WaveOne Oneshape	R R CR	Distilled water	Reciproc and Waveone > Oneshape
**Silva *et al.***	Mn premolar	60	ProTaper Universal ProTaper Next WaveOne Reciproc	CR CR R R	Distilled water	ProTaper Universal > ProTaper Next, WaveOne and Reciproc
**Topçuoglu *et al.***	Mn premolar	60	Vortex Blue K3XF Reciproc ProTaper Next	CR CR R CR	Distilled water	K3XF and Reciproc > Vortex Blue and ProTaper Next
**Kuştarcı *et al.***	Mn premolar	90	Reciproc Revo-S Twisted File Adaptive	R CR AM	Distilled water	No significant difference

Mn, mandible; Mx, maxillar; MA, adaptive movement; RC, continuous rotation; R, reciprocation.

**Table 3:** Summary of characteristics of the studies about dentinal defects.

**Author**	**Type of Samples**	**Number of Canals**	**File Used**	**Kinematics**	**Methods**	**Results**
**Coelho *et al.***	Mn molar	160	ProFile TRUShape WaveOne Gold	CR CR R	Led transillumination	No significance difference
**Ustun *et al.***	Mn incisor	120	K-File ProTaper Universal ProTaper Next Reciproc	MI R and CR CR R	SEM	No significance difference
**Gergi *et al.***	Mn molar	180	Reciproc WaveOne Twisted File Adaptive	R R CR and R	SEM	Reciproc > WaveOne and Twisted File Adaptive
**Jalali *et al.***	Mn premolar	100	Reciproc ProTaper Universal Mtwo	R CR CR	SEM	Mtwo and ProTaper Universal > Reciproc
**Karataş *et al.***	Mn incisor	75	ProTaper Universal ProTaper Next WaveOne Twisted File Adaptive	CR CR R AM	SEM	ProTaper Universal and WaveOne > ProTaper Next and Twisted File Adaptive
**Karataş *et al.***	Mn incisor	105	Twisted File Adaptive	CR/R/AM	SEM	RC and Adaptive Movement > R
**Aydin *et al.***	Mn premolar	70	Reciproc WaveOne Twisted File Adaptive	R R AM	SEM	No significance difference
**De-Deus *et al.***	Mn molar	20	ProTaper Next Twisted File Adaptive	CR R	µCT	No formation of dentinal defects
**Zhou *et al.***	Mn premolar and molar	280	WaveOne Protaper Universal Twisted File Twisted File Adaptive	R CR CR AM	SEM	WaveOne and ProTaper Universal > Twisted File and Twisted File Adaptive
**De-Deus *et al.***	Mn molar	30	Reciproc WaveOne BioRace	R R CR	µCT	No significance difference
**Bürklein *et al.***	Mn incisor	100	Mtwo ProTaper Reciproc WaveOne	CR CR R R	SEM	WaveOne and Reciproc > Mtwo and ProTaper

Mn, mandible; Mx, maxillar; MI, manual instrumentation; AM, adaptive movement; CR, continuous rotation; R, reciprocating; CT, computed tomography; CBCT, cone beam computed tomography; SEM, scanning electron microscopy.
